# Saturated anacardic acid preserves root dentin collagen under cariogenic challenge

**DOI:** 10.1007/s00784-026-07178-y

**Published:** 2026-09-28

**Authors:** Bruna Leis Endres, Isabela Monici Silva, Luiz Antonio Soares Romeiro, Caio Matheus Ferreira, Antônio Djalma Santos, Sebastião William da Silva, Márcio Botelho de Castro, Leandro Augusto Hilgert, Luana Wouk, Andressa Souza de Oliveira, Fernanda Cristina Pimentel Garcia, Naile Dame-Teixeira

**Affiliations:** 1 Department of Dentistry, School of Health Sciences, University of Brasília (UnB), Brasília, Brazil; 2 Center for Tropical Medicine, University of Brasília (UnB), Brasília, Brazil; 3 Institute of Physics, University of Brasília (UnB), Brasília, Brazil; 4 Laboratory of Veterinary Pathology and Forensics, University of Brasília (UnB), Brasília, Brazil; 5 Department of Pharmacy, University of Brasília (UnB), Brasília, Brazil

**Keywords:** Collagen, Dental materials, Dentin, Fourier transform infrared spectroscopy, Polarization microscopy, Root caries

## Abstract

**Objective:**

We aimed to evaluate changes in the structure and organization of root dentin collagen in bovine incisors pre-treated with an anacardic acid derivative (LDT11) following an in vitro cariogenic challenge, thereby contributing to understanding of dentin matrix biology and biomodification strategies for root caries management.

**Materials and methods:**

Bovine root dentin specimens were demineralized and pre-treated with LDT11 (100 µg/mL) or distilled water (control). Samples were then subjected to a cariogenic challenge for 7 days. Histological sections were stained with Picrosirius and analyzed by polarized light microscopy to assess collagen organization and residual collagen area. Additional analyses were performed using Fourier Transform Infrared Spectroscopy in Attenuated Total Reflectance mode (FTIR-ATR) and thermogravimetric analysis (TGA).

**Results:**

LDT11-pretreated samples showed a significantly greater residual collagen area compared to controls (*p* < 0.01), with preservation of a dense and organized fibrillar network. FTIR-ATR analysis revealed spectral changes consistent with hydrogen bonding and collagen biomodification. Principal component analysis demonstrated clear separation between treated and untreated groups. In contrast, TGA showed no significant differences in total collagen mass loss, suggesting a localized effect of LDT11 at the superficial dentin layer.

**Conclusion:**

LDT11 promotes preservation of the dentin collagen matrix under cariogenic challenge and exhibits potential as a collagen biomodifier through chemical interaction with the organic matrix. Its effects appear to be primarily surface-oriented, contributing to structural stability without altering collagen mass.

**Clinical relevance:**

Potential of LDT11 as a dentin-interacting agent that can help control collagenolytic degradation as an adjunct for managing root caries lesions.

**Supplementary Information:**

The online version contains supplementary material available at https://doi.org/10.1007/s00784-026-07178-y.

## Introduction

It has been discussed that root caries lesions begin with dentin demineralization, followed by degradation of the collagenous matrix [1, 2]. In the early stages of lesion formation, mineral loss partially preserves the organization of collagen fibers, which act as a scaffold for microbial colonization. Over time, collagenolytic enzymes degrade the exposed collagen, compromising its structural integrity [1–4]. Among these enzymes, matrix metalloproteinases (MMPs) have a central function, as they are activated by acidic conditions, inflammatory cytokines, and oxidative stress [1, 5, 6]. In addition, cathepsins and other host-derived proteases contribute to collagen solubilization, further accelerating dentin matrix degradation [7]. It is also likely that collagenolytic proteases produced by the cariogenic biofilm itself participate in this stage of lesion development [7].

Given the limitations of conventional root caries treatments, largely related to the composition of root dental tissues, biomodifying compounds have emerged as adjuncts to non-invasive therapies. Some agents, for instance, could form crosslinks with collagen in the organic matrix of dentin or cementum, enhancing structural stability and improving both mechanical and biochemical properties [8]. This same crosslinking approach has been applied to enhance the durability of the hybrid layer in adhesive restorations [9–12]. In this context, natural compounds such as green tea polyphenols, proanthocyanidins, and derivatives of cashew nut shell liquid (CNSL), including cardanol, cardol, and anacardic acid (AA), have gained attention due to their effectiveness, lower cytotoxicity, and sustainable origin [12–15]. AA, the major component of CNSL, exhibits inhibitory activity against MMPs, along with antimicrobial and anti-collagenase effects, and is biocompatible with dental pulp stem cells [16–19].

In our previous studies, a saturated derivative of AA (LDT11) showed potential interaction with dentin collagen, as well as significant modulation of the microbial profile in complex biofilms. In an in vitro root caries model using complex biofilms under sucrose cycling, LDT11 altered biofilm biological processes and metabolic functions, particularly when applied as a pretreatment (prior to biofilm formation on dental specimens), promoting changes associated with metal binding and the regulation of metabolic pathways related to enzymatic activity [20]. RNA-seq analysis of these biofilms revealed that, among the 32 annotated genes encoding U32 family collagenases, predominantly belonging to the genus *Prevotella*, none showed detectable expression in biofilms pre-treated with LDT11, indicating complete transcriptional inhibition [20]. This selective suppression of microbial collagenase gene expression, combined with its ability to interact with calcium and dentin collagen, reinforces the potential of LDT11 as a multifunctional bioactive agent for preventing degradation of the organic matrix in root caries lesions. Therefore, this study aimed to evaluate changes in the structure and organization of root dentin collagen in bovine incisors pre-treated with LDT11 following an in vitro cariogenic challenge, contributing to the understanding of dentin matrix biology and biomodification strategies for root caries management.

## Materials and methods

### Sample preparation

Roots of bovine incisors were used for dentin sample preparation (Supplementary Fig. 1). To measure Fourier Transform Infrared Spectroscopy in Attenuated Total Reflectance Mode (FTIR-ATR), samples were prepared using a high-precision cutting machine, resulting in two approximately 500 μm-thick and 8 mm wide samples from each root from bovine incisor. Longitudinal sections were made along the long axis of the tooth, obtained from mesial and distal portions, excluding regions of the root canal and root cementum. The samples were not subjected to additional grinding or polishing to avoid further loss or alteration of the collagen structure.

For cariogenic challenge experiments, dentin discs were sectioned from the root surface using a double-sided diamond disc (KG Sorensen) on a handpiece (Supplementary Fig. 2). Specimens were decontaminated in 0.5% chloramine solution, rinsed with distilled water, and immediately demineralized and subsequently pretreated with the experimental solutions.

### Experimental groups

The compounds used in this study were saturated anacardic acid (LDT11) and cardanol (LDT409) [19]. Both were extracted from CNSL, purified, and chemically modified at the Laboratory for the Development of Therapeutic Innovations, Tropical Medicine Center, University of Brasília, Brazil. Details regarding the chemical design, structure, and preparation of LDT11 were previously described by de Souza et al. (2018) (Supplementary Fig. 3) [21]. Solutions were prepared from isolated and dehydrated compounds, initially reconstituted in 1 mL of 95% ethanol and subsequently diluted to a final concentration of 7 mg/mL, with a total volume of 1 mL.

Specimens for each experiment were randomly allocated using a random numbers table generated on the “openepi.com” website for the allocation of prepared dentin blocks by outcome and by group into the following groups (*n* = 3 per group): cardanol (LDT409 – positive control only for the FTIR-ATR experiment); saturated anacardic acid (LDT11) 100 µg/mL; untreated control (distilled water).

Root bovine specimens were demineralized with 10% phosphoric acid for 30 min to expose the dentin organic matrix following the protocol established by Wang et al.[15], pre-treated according to the respective groups and rinsed with distilled water to remove unbound compounds (Supplementary Fig. 4). All experiments were performed in triplicate.

### Fourier transform infrared (FTIR) spectrometer with attenuated total reflectance (ATR) module

In this experiment, after demineralization, the samples were completely immersed in 1 mL of their respective treatment solution for 72 h [22]. The experimental setup was designed as a static model, with specimens remaining immersed in the same treatment solution throughout the incubation period. Afterward, the specimens were thoroughly rinsed with distilled water and vacuum-dried to prevent interference in the OH spectral region. The interactions between the compounds (LDT11 and LDT409) and demineralized bovine dentin were analyzed using Fourier-Transform infrared (FTIR) spectroscopy with an attenuated total reflectance (ATR) accessory on a Bruker Vertex 70 spectrometer. For each measurement, 96 scans were averaged at a resolution of 4 cm^− 1^. The same number of scans was averaged the background signal before each measurement, which was subsequently subtracted.

To assess the correlation between different groups and the multiple spectra, as well as respective peaks obtained from each sample, a Principal Component Analysis (PCA) analysis was performed using Minitab^®^ 17.1.0. The calculation of the areas of peaks in Amide I was performed using Fitik software version 0.9.8 with the Voigt function. Analysis of Variance (ANOVA) was used to compare groups. The power of the study was performed by using the mean difference of the Amide I peak and considering a 95% two-tailed confidence interval using Openepi tool (https://www.openepi.com/Power/PowerMean.htm).

### Collagen preservation after cariogenic challenge

#### Pretreatment of dentin with anacardic acid LDT11 and carious lesion formation

For the following experiments, after demineralization, the samples were completely immersed in 1 mL of the respective treatment solution for 72 h [22]. Afterward, the specimens were thoroughly rinsed with distilled water and stored in 50 mM PBS for 48 h prior to inoculation for in vitro carious lesion formation.

An in vitro caries lesion formation protocol was performed using *Streptococcus mutans* UA159. Dentin samples were cultured in a 48-well plate with 200 µL of overnight inoculum and 800 µL of BHI broth, and incubated at 37 °C for 24 h. Subsequently, the specimens were transferred to a new plate containing fresh BHI medium, and sucrose cycling was initiated to simulate a cariogenic environment: the medium was supplemented with 1% sucrose during the sucrose-exposure phase (6 h) and replaced with pure BHI broth during the non-exposure phase (24–48 h). The cariogenic challenge was conducted for 7 days, including 3 days of sucrose cycling (Supplementary Fig. 5).

At the end of the caries lesion formation period, the dentin discs were aseptically removed from the wells, and the formed biofilm was scraped off. The samples were then placed in sterile microcentrifuge tubes containing 50 mM PBS and subjected to ultrasonic bath agitation for 15 min to detach and remove any remaining biofilm.

#### Histological processing and picrosirius staining

Immediately after biofilm removal, the samples designated for Picrosirius staining were fixed in 10% formalin and stored in Falcon tubes. After the fixation period, the samples were rinsed in running water and stored in 50 mM PBS until the start of decalcification and histological processing for evaluation under polarized light microscopy.

Decalcification was performed using a 5% nitric acid solution diluted in 10% formalin to improve tissue structural preservation [23, 24]. Upon completion of decalcification, the samples underwent a second fixation in 10% formalin, followed by histotechnical processing in an automatic vacuum processor (Tissue-Tek VIP 5 Jr., Sakura).

After paraffin infiltration, the specimens were embedded in blocks, and histological sections were obtained and mounted on labeled glass slides. Staining was performed according to the instructions of the Picrosirius-Hematoxylin kit (Histokit – Easypath). After staining, the slides were dehydrated, cleared, and mounted with a synthetic mounting medium (ERV-MOUNT) and glass coverslips for subsequent microscopic analysis.

#### Polarized optical light microscopy

Picrosirius-stained slides were analyzed using a polarized optical light microscope (Olympus BX50) equipped with a coupled MOTICAM S6 camera, using a 10× objective, a phase 3 polarizing filter, and intermediate light intensity. Images were captured at maximum brightness, with the polarizer and analyzer set at 45° to each other. For each histological section, three representative random areas of dentin facing the root canal center were selected for standardization. Although randomly chosen, efforts were made to include areas corresponding to each wall of the root structure.

The acquired images were adjusted using Photoshop software to enhance sharpness and include a scale bar (100 μm). Subsequently, images were processed using ImageJ software to delineate the corresponding root dentin area. Based on these delineated images, the residual collagen area of the samples was calculated (Supplementary Fig. 6). Data were analyzed using Prism for macOS (Version 9.5.0). After normality testing, the Kruskal–Wallis test followed by Dunn’s multiple comparison test was performed, with statistical significance set at *p* < 0.05.

#### Thermogravimetric analysis (TGA)

After the removal of the biofilm, samples were measured using thermogravimetric analysis (TGA) to measure the amount of collagen present on them. For that, samples were pressed and manually ground to obtain a powder that can be measured using ceramic crucibles. In TGA analysis, normalization is performed based on the sample’s initial mass, which is measured prior to the experiment. Measurements were performed using a TGA/DSC 3 + STARe System from Mettler-Toledo. All measurements were conducted in inert nitrogen atmosphere, with gas flow rate of 20 mL/min. The system was heated with a heat ramp of 10 °C/min from room temperature until 850 °C. A triplicate of all samples was performed to ensure statistical significance. For simplicity a representative curve of the triplicate is shown in data.

The amount of collagen was measured by calculating the mass loss over the temperature range of 220 °C to 430 °C, related to the collagen thermal degradation [25, 26]. This was performed by numerical integration of the first derivative of TGA with respect to time (DTG) in the corresponding temperature range of collagen degradation [27]. Uncertainty was calculated based on the standard deviation of the triplicate measurements. Lastly, the data were subjected to statistical analysis using the software (Prism for MacOS Version 9.5.0, employing the Kruskal–Wallis test with multiple comparisons using Dunn’s test after normality testing, considering differences significant at *p* < 0.05.

## Results

### Interaction between LDT11 and collagen

**Fig. 1:**
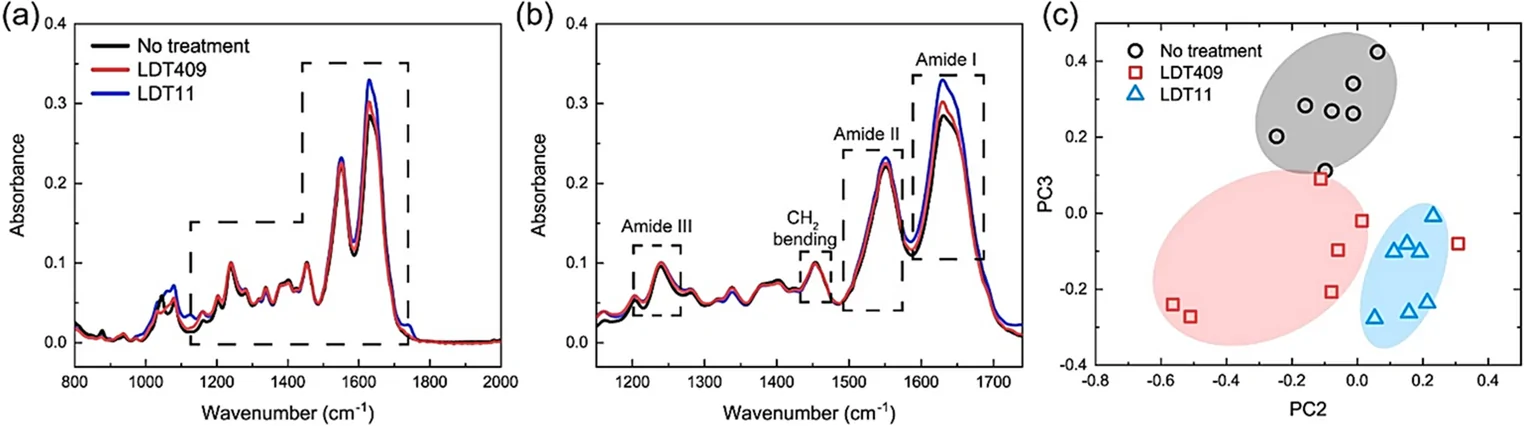
Material characterization of bovine dentin treated with LDT11 by FTIR-ATR analysis. (**a**) Representative FTIR-ATR spectra of untreated dentin, positive control (LDT409), and LDT11-treated samples. (**b**) Expanded spectral region highlighting subtle alterations in collagen-associated bands, including Amide I, II, and III, as well as CH₂ bending vibrations related to the structural organization of collagen. (**c**) Principal component analysis (PCA) of the FTIR-ATR data demonstrating clear discrimination among the experimental groups, with LDT11-treated samples exhibiting a spectral profile more closely associated with the LDT409 positive control than with untreated dentin

The results of the FTIR-ATR are summarized in Fig. 1a. The spectra analysis focused on changes in the Amide I, II, and III regions, as well as the CH₂ bending regions, all of which reflect the structural characteristics of collagen. These bands correspond to distinct peptide bond vibrations: specifically, C = O stretching (Amide I), N–H bending coupled with C–N stretching (Amide II), and the combined C–N, N–H, and CH₂ vibrations (Amide III) [28] (Fig. 1b).

The interaction of LDT11 and the positive control (LDT409) with the collagen framework of bovine dentin induce two main effects: a slight reduction in the peak intensity at ~ 1400 cm^− 1^, and the appearance of new peaks in amide I and amide II regions. Within the former region, an increase in intensity at 1630 cm^− 1^ was also observed. Regarding the Amide III peak (~ 1239 cm^− 1^), LDT409 treatment led to a clear increase in intensity (Fig. 1a). Furthermore, both treated groups exhibited alterations in the 1000–1200 cm^− 1^ range, including peak broadening and increased intensity. A notable shoulder emerged at ~ 1120 cm^− 1^ for the group treated with AA.

Given these subtle yet consistent spectral changes, principal component analysis (PCA) was employed to further discriminate between the groups. The results, shown in Fig. 1c, reveal a clear separation among the groups, indicating a positive correlation between the LDT409 positive control group and LDT11 test group. Moreover, the LDT11 treated groups exhibit a different correlation pattern compared with untreated samples. Collectively, these findings suggest that the subtle spectral alterations, together with the PCA discrimination, indicate that LDT11 may promote biomodification of the collagen matrix predominantly through intermolecular interactions, particularly hydrogen bonding, with its interaction with collagen being clearly discernible through multivariate analysis. Based on these results, only LDT11 was selected for subsequent cariogenic challenge experiments.

### Preservation of the superficial dentin collagen matrix

**Fig. 2:**
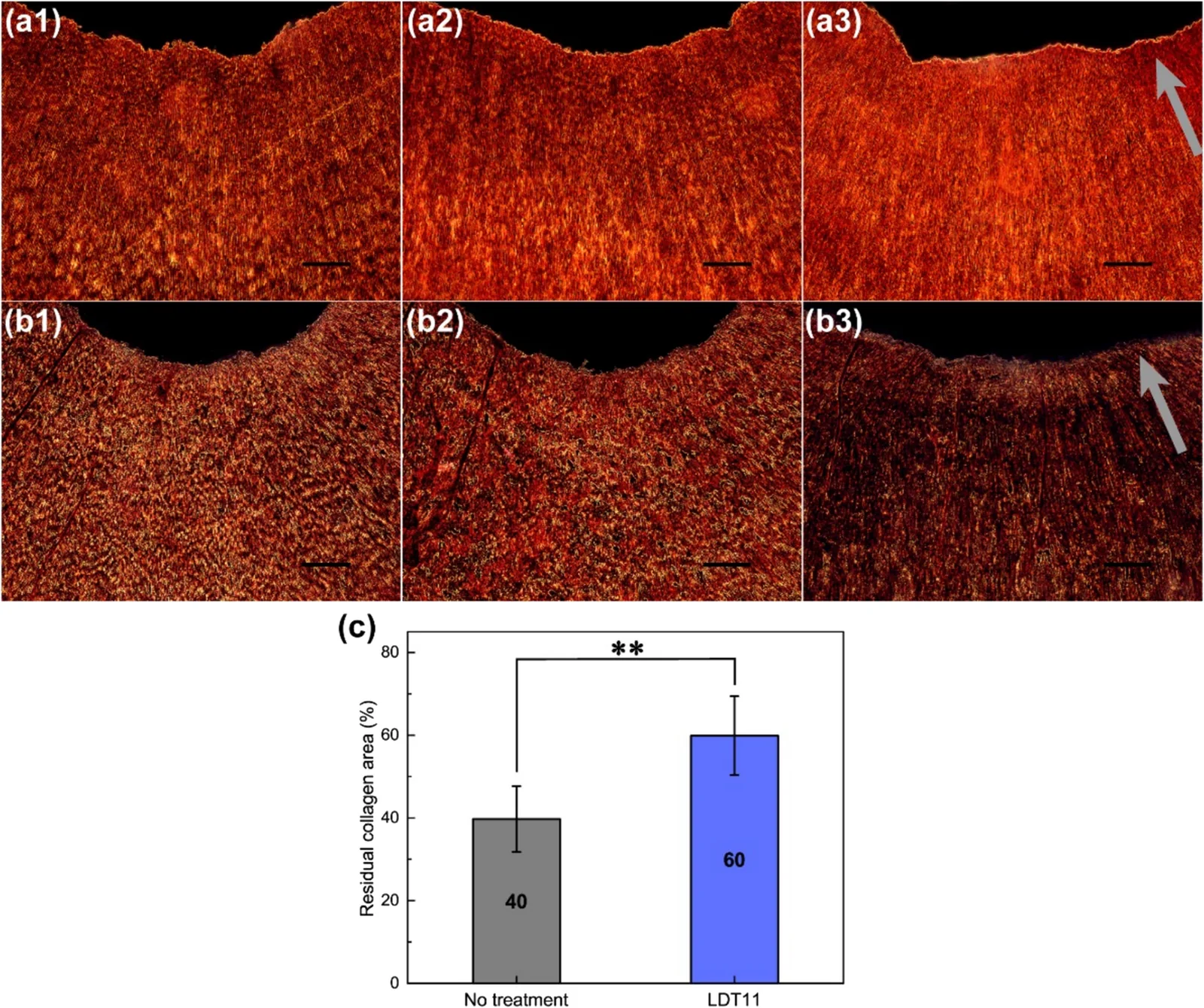
Polarized optical microscopy analysis for identification and quantification of collagen fibers after the cariogenic challenge. Representative images of samples (1–3) from the (a1–3) LDT11-pretreated group and (b1–3) untreated control group. LDT11-treated samples exhibited enhanced preservation of root collagen fibers, characterized by an organized and well-oriented structure with predominant orange-red birefringence, indicative of mature collagen. In contrast, untreated samples showed disorganized fibers, matrix voids, and birefringence ranging from yellow to green, consistent with collagen degradation. All images were obtained under crossed polarizer conditions (analyzer positioned at 45° relative to the polarizer). The continuity of the collagen matrix and the presence of brighter regions, indicated by arrows, suggest the preserved collagen zone area. Images were acquired facing the root canal. Scale bars = 100 µm. (c) Quantitative analysis of residual collagen surface area (%) comparing LDT11-treated and untreated groups. The LDT11 group exhibited a significantly greater residual collagen area after the cariogenic challenge (**p < 0.01; Kruskal–Wallis test). Data are presented as mean ± standard deviation

Microscopy with Picrosirius staining images from the LDT11-pretreated group demonstrated a predominance of type I collagen in the organic matrix of the dentin. This was evidenced by the predominance of red and yellow hues in 4 μm histological sections of thickness. A brighter band was observed in the region corresponding to preserved collagen zone, which represents a more organized area of the collagen matrix. Additionally, a uniform fibrillar network was noted, indicating a dense and closely packed organic matrix (Fig. 2a(1–3)).

In the untreated group, depicted in Fig. 2(b(1–3)), the preserved collagen zone region was not clearly identifiable in some images. The dentin structure in this group appeared more disorganized, with evidence of structural loss and visible discontinuities in the collagen network. Furthermore, the organic matrix exhibited a less dense, more loosely arranged organizational pattern (Fig. 2b), differing from both the LDT11 group and the morphological pattern considered normal for bovine dentin [23, 24].

Samples pre-treated with the LDT11 solution showed a greater residual collagen area compared to the untreated group, with higher mean percentage values (Fig. 2c). In the LDT11 group, the collagen fiber area fraction was (59.9 ± 9.5)% (mean ± standard deviation), significantly higher than that of the untreated group (*p* < 0.01). These findings indicate that LDT11 reduced collagen degradation and preserved it under cariogenic challenge, as evidenced by the maintenance of the characteristic structural and organizational pattern of the organic matrix.

### Relative collagen mass quantity

**Fig. 3:**
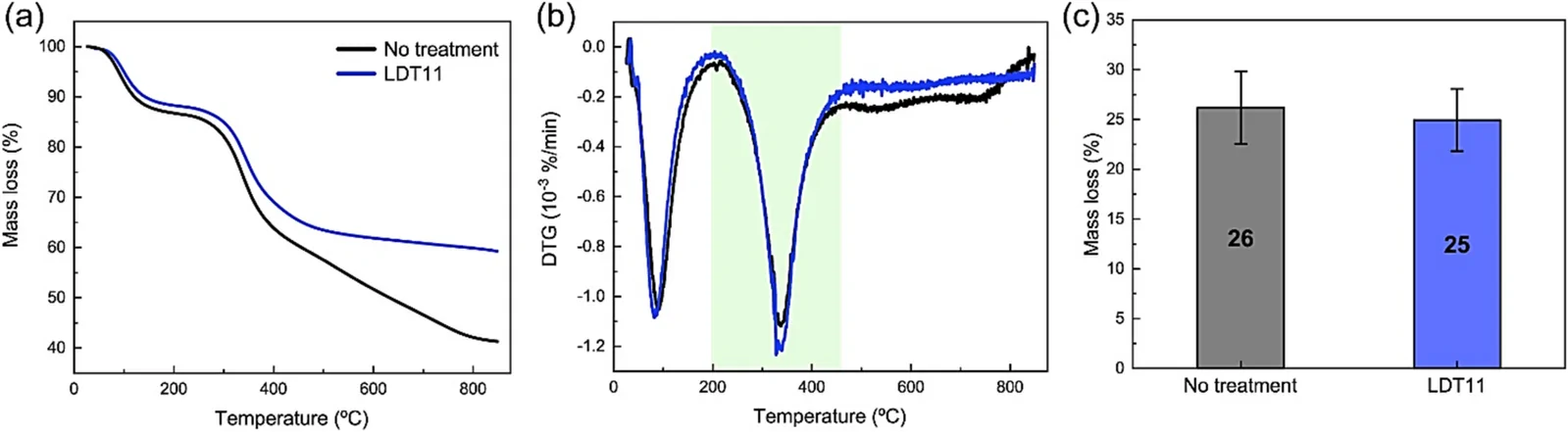
Thermogravimetric analysis (TGA) of bovine dentin samples evaluating material stability and degradation. (**a**) TGA curves comparing the thermal behavior of LDT11-treated and untreated control groups. (**b**) Derivative thermogravimetric (DTG) curves showing two main mass loss events: an initial phase associated with water evaporation and volatile compounds, followed by a second phase predominantly related to collagen thermal degradation, highlighted in green (220–430 °C). Both groups exhibited similar thermogravimetric profiles, with comparable peak patterns and areas under the curves, indicating equivalent overall mass loss. (**c**) Relative collagen mass loss (%) in untreated and LDT11-treated samples, analyzed using the Kruskal–Wallis test. No statistically significant differences were observed between groups. Data are presented as mean ± standard deviation

Thermogravimetric analysis allowed the observation of the behavior of the groups, highlighting the regions of interest as described by Cucos et al. (2014) [29] (Fig. 3a–b). The 220–430 °C range was selected as it predominantly corresponds to the thermal degradation profile of collagen [25, 26]. In the LDT11 group, the collagen fiber area fraction was (24.9 ± 3.1)% (mean ± standard deviation). No statistically significant differences were observed in the percentage of relative collagen mass loss, suggesting that the evaluated compound did not significantly permeate into the bulk collagen network as a whole in terms of total mass under the in vitro cariogenic challenge (Fig. 3c). Overall, both the LDT11-pretreated and untreated control groups exhibited similar thermogravimetric profiles, characterized by continuous mass loss over the temperature range of 30 to 850 °C. The peaks and the area under the curve were comparable, indicating that the total amount of mass lost was equivalent between the groups, as can be seen from the DTG data of Fig. 3(b).

## Discussion

The search for compounds that can serve as adjunctive therapies in the management of root caries lesions, particularly by inhibiting collagenolytic activity and preserving the dentin matrix, represents a promising and still emerging field of translational research with potential clinical applicability. Some natural agents were shown to have the potential to biomodify dentin by forming exogenous crosslinks between molecules and type I collagen, the predominant component of root dentin [8]. This process may enhance structural stability and improves the mechanical and biochemical properties of the tissue [30]. Preservation of dentin collagen may delay the progression of root caries lesions, as the integrity of this matrix acts as a protective barrier, hindering enzymatic degradation and the advancement of demineralization. In this context, LDT11, an anacardic acid (AA) compound derived from byproducts of the food industry [31], stands out due to its multifunctionality and biocompatibility. It has demonstrated potential to stimulate the proliferation and differentiation of dental pulp stem cells, inhibit bacterial collagenase activity [19], among other biological functions. These properties reinforce its relevance as a candidate for protecting collagen against the progression of root caries lesions [19].

This study demonstrated alterations in the organic matrix potentially compatible with interactions between collagen and the compound, as evidenced by FTIR-ATR analysis. These observed spectral modifications suggest a possible interaction between the LDT11 and dentin. This interpretation is reinforced by PCA analysis, which demonstrated clear separation between the LDT11-treated group and the untreated control, indicating that the spectral profile of LDT11-treated samples closely resembles that of the positive control. Notably, the interaction between cardanol and the dentin collagen matrix has been previously established, supporting its use as a positive control [12, 14]. The concentration of 100 µg/mL used in this protocol was selected based on its effectiveness in preserving the organic matrix and inhibiting collagenase-related genes (U32 family). So, at this specific concentration, LDT11 appears to achieve a favorable balance between biological efficacy and biocompatibility. In vitro studies suggest that concentrations around 100 µg/mL are sufficient to exert anti-collagenolytic activity, modulate microbial metabolism, and promote greater stability of the dentin organic matrix, indicating that this concentration range may be suitable for both professional and home-use products [19–21, 31]. Incorporation of LDT11 into topical delivery systems could enable sustained action on exposed dentin, facilitating partial penetration into the collagen matrix and protection against enzymatic degradation. Additionally, an important advantage of LDT11 lies in its origin from cashew nut shell liquid, an abundant, renewable, and low-cost material derived from the Brazilian agroindustry. This availability supports large-scale production, reduces synthesis costs, and aligns with sustainable approaches to the development of effective and accessible dental biomaterials.

This study provides the first chemical evidence of an interaction between AA (LDT11) and dentin using FTIR-ATR, highlighting its role as a possible collagen biomodifier and reinforcing the crosslinking-related effects. Previous studies with plant-derived polyphenols have reported reduced intensity around ~ 1400 cm⁻¹ indicating dehydration and collagen crosslinking-like behavior attributed to hydrophobic interactions, which enhances structural stability. Similar patterns were observed in this study for both AA (LDT11) and cardanol (LDT409, used here as a control in the FTIR-ATR experiment). Additional indicators, such as reduced Amide II intensity (~ 1550 cm⁻¹), the formation of spectral shoulders, and increased Amide I intensity (~ 1630 cm⁻¹), further suggest hydrogen bonding and collagen biomodification [15, 32–34]. Changes in the 1000–1200 cm⁻¹ region may indicate either collagen degradation or chemical interactions. Given the experimental conditions, hydrogen bonding between the AA hydroxyl groups and collagen amine groups is the more likely explanation. This interpretation is supported by Raman spectroscopy studies, which have also demonstrated cardanol’s potential interaction [35].

Ethanol was used here only as the initial solvent for the reconstitution of LDT11 and LDT409, as previously described [19], resulting in a low final concentration in the pre-treatment solutions. Therefore, the amount of ethanol present during treatment was minimal, as also reported in our previous study [19]. The same ethanol concentration was previously used in cytotoxicity assays of LDT11 and LDT409 using human dental pulp stem cells, in which low cytotoxicity (< 20%) and no detrimental effects on cell viability or growth were observed under the tested conditions. In addition, ethanol was evaluated as a solvent control at the same concentration used in the present study in our anti-collagenolytic activity assays, with no effect attributable to ethanol [19]. The FTIR results provide further support that the spectral changes observed in the present study were more likely related to the treatment agents than to ethanol itself. Both LDT11 and LDT409 were prepared using ethanol, yet distinct spectral profiles were observed between the treatment groups, with clear separation of the groups in the PCA. This pattern suggests that the molecular interactions detected by FTIR were associated with the distinct chemical characteristics of the treatment agents rather than with the common solvent. Thus, although a contribution of ethanol to the interaction with the hydrophilic collagen matrix cannot be completely excluded, the low final ethanol concentration, the absence of an effect in the solvent control, the previous cytotoxicity findings, and the distinct FTIR responses observed for compounds prepared in the same solvent collectively indicate that ethanol was unlikely to be responsible for the molecular changes observed in the present study.

Microscopy findings further support this hypothesis that LDT11 interacts with the dentin collagen matrix, potentially enhancing its stability against proteolytic degradation. Pretreatment with the compound reduced degradation of the organic matrix, as evidenced by an increase in the residual collagen area on the surface of treated samples. Picrosirius staining revealed greater preservation of collagen fibers on LDT11-pretreated surfaces, indicating that the compound contributed to the preservation of collagen fibers at the surface under an in vitro cariogenic challenge. Picrosirius staining combined with polarized light microscopy is considered a gold standard for the identification and quantification of collagen fibers [36, 37], with high sensitivity and broad application in studies of development, repair, and degradation of mineralized tissues [38–40]. In the stained histological samples, a predominance of red and yellow-green hues was observed under polarized light, consistent with the literature. The superficial dentin region exhibited intense birefringence, suggesting a more organized collagen matrix [41]. The LDT11-pretreated group showed greater preservation of root collagen fibers, which remained organized, well-oriented, and predominantly stained orange-red, consistent with mature collagen [23, 24]. This group showed a significantly higher residual collagen area than the control, with disorganized fibers, matrix voids, and color variations from yellow to green, indicative of structural degradation [42]. The ability of LDT11 to preserve a dense and organized fibrillar network under cariogenic challenge may create a biological barrier capable of slowing lesion progression and limiting the diffusion of harmful microbial byproducts toward the pulp tissue.

Other than the crosslinking formation, an explanation for collagen protection is the inhibition of collagenases during second stage of caries lesion formation, reducing collagen degradation. Recent findings from our research group corroborate these results. In a complex cariogenic biofilm model, LDT11 modulated microbial metabolism by inhibiting the expression of U32 collagenase genes, which are responsible for collagen cleavage and the virulence of proteolytic species [20]. Additionally, the compound reduced bacterial gelatinase activity, as evidenced by decreased degradation of collagen and gelatin substrates [20]. These findings suggest that LDT11 interferes with microbial and enzymatic pathways involved in proteolytic degradation, possibly through metabolic modulation and direct inhibition of metal-dependent enzymes.

Beyond its well-established antimicrobial, biological, and therapeutic properties [43], AA has emerged as a potent inhibitor of MMPs [16, 17, 44, 45]. AA is thought to inhibit MMP activity through chelation of the catalytic zinc ion at the enzyme active site [17]. In addition to directly suppressing enzymatic activity, AA modulates MMP regulation at the transcriptional level by downregulating gelatinases and their endogenous activators (MMP-14 and EMMPRIN), while upregulating endogenous inhibitors such as RECK and Spry2 [16]. Although the reported inhibitory effects of AA on MMPs vary across studies, its activity appears consistently dose-dependent [16]. Omanakuttan et al. [17] demonstrated significant inhibition of MMP-2, a key enzyme involved in dentin collagen degradation, at concentrations as low as 1 µg/mL (3 µM), reaching 98% inhibition at 200 µM. Comparable inhibitory effects were observed for MMP-9. Similarly, Nambiar et al. [16] reported inhibition of both MMP-2 and MMP-9 at concentrations ≥ 5 µM. In an in vitro erosion model, Silveira et al. [46] showed that saturated AA reduced dentin tissue loss more effectively than other natural phenolic compounds and sodium fluoride (NaF), with significant protection observed at 100 µmol/L. The translational potential of AA for oral healthcare applications has also been explored. Araújo et al. [47] evaluated AA-containing mouthrinses for the management of dentin erosion and carious lesions and reported no genotoxic effects at 9.375 µg/mL, supporting its short-term biocompatibility. Nevertheless, further in vivo and clinical studies are needed to establish its long-term safety and therapeutic efficacy.

While Picrosirius microscopy demonstrated substantial surface preservation, TGA did not reveal significant differences. TGA analysis, which provides information on material stability and degradation [48], did not reveal significant differences in the relative collagen mass between treated and control groups. This may be explained by the localized action of LDT11, likely restricted to the superficial micrometers of dentin. Although TGA did not reveal statistically significant differences in total collagen mass loss, this result is consistent with a predominantly superficial action of LDT11, since TGA evaluates the bulk mass of the powdered sample rather than only the treated surface layer. Such an effect may be sufficient to protect the hybrid layer (~ 0.5–4 μm), but insufficient to significantly alter the total mass within the entire sample volume [49–52]. This hypothesis of hybrid layer protection in restorative treatments deserves future studies and should include bond strength or interfacial nanohardness tests to determine whether this superficial preservation translates into clinically relevant effects.

Although previous investigations demonstrated LDT11 excellent biocompatibility [19], preliminary observations suggest that the viability of dental pulp stromal cells (DPSC) can reduce when co-cultured with dentin slabs (data not shown), meaning that the presence of calcium-rich substrates such as dentin may influence cellular responses, a hypothesis that warrants further investigation. It means that LDT11 appears to be biologically safe for DPSC, however, in calcium-rich environments it can present different results, because its natural chemical structure allows it to bind to the dentin surface through physicochemical interactions. Importantly, our TGA findings suggest that the interaction of LDT11 with dentin is predominantly superficial and does not alter the total organic mass of bulk dentin. Due to its affinity for calcium ions within dentin, the compound tends to remain concentrated at the application site, primarily on the superficial layers of the substrate. This localized retention may limit diffusion toward the pulp tissue, thereby reducing the risk of cytotoxic effects on deeper tissues. In addition, the ability of LDT11 to form hydrogen bonds and interact with calcium may promote retention of the agent at the dentin interface, potentially limiting its diffusion toward the pulp tissue and reducing cytotoxic effects at clinically relevant concentrations. As in the absence of calcium LDT11 did not exhibit cytotoxicity in DPSC, it seems that only calcium-mediated interactions may play a role in its cytotoxicity.

Considering that the action of LDT11 is highly localized to the superficial micrometer-scale layers of dentin, this restricted penetration suggests that, under clinical conditions, the compound would be unlikely to reach the pulp tissue at cytotoxic concentrations, acting selectively at the site where hybrid layer protection is required. It should also be considered that in vitro dentin disc cytotoxicity models frequently omit physiological factors present in vivo, particularly the positive pulpal hydrostatic pressure that limits the diffusion of substances toward the pulp tissue. Moreover, the preservation of a dense and organized fibrillar network promoted by LDT11 may itself function as an additional physical barrier, reducing dentin permeability over time and potentially minimizing the initial cytotoxic risks reported in passive diffusion models. Therefore, if dentin permeability remains low after treatment, the cytotoxicity observed against pulp cells in vitro may have limited clinical relevance. Nevertheless, the implications of these findings for the translational applicability and long-term safety of the product still require further investigation.

The use of bovine incisors may be considered a limitation of this study. However, they are widely used as substitutes for human teeth in adhesion studies involving restorative materials and dental structures. Schilke et al. (2000) demonstrated that, in terms of tubule number and diameter, there are no significant differences between human and bovine coronal dentin [53]. In the present study, the induction of in vitro root caries lesions via sucrose cycling enabled the simulation of a cariogenic environment and the assessment of the effectiveness of LDT11 in preserving collagen. Despite these limitations, the localized collagen protection conferred by LDT11 is clinically relevant, considering that the quality of the hybrid layer in caries-affected substrates is often compromised and directly associated with the longevity of restorations [54]. Furthermore, the incorporation of AA into universal adhesive systems does not appears to impair their physicochemical and mechanical properties, while also providing antimicrobial and antibiofilm activity [55]. The findings of this study reinforce the potential of LDT11 as a dentin-interacting agent that can help control collagenolytic degradation and, consequently, manage root caries lesions [19].

One can argue that the treatment period may be limited in terms of clinical applicability Based on previous studies testing other compounds as cross-links in similar conditions, we decided to keep our samples during 72 h in contact with the compounds [20, 22]. After showing this initial positive response, we understand that next steps can involve testing shorter clinically relevant time of application. Although qualitative µCT observations from experiments performed under comparable conditions indicated predominantly superficial mineral loss [20], the depth of demineralization was not quantified in the present study and may vary according to the experimental conditions. Nevertheless, this limitation is unlikely to substantially affect the comparative interpretation of our findings, as multiple biological replicates were included and dentin specimens were randomly allocated across experimental groups, helping to distribute inherent biological variability among conditions. Another potential limitation is the mechanical removal of biofilms after lesion formation that could have disturbed the collagen, however, it was performed carefully and gently, finalized by ultrasonic agitation, to minimize potential damage to the hard tissue of the specimens and to minimize any effect in the experiments.

Next steps include evaluating elution and permeability over time are needed to accurately map the kinetics of LDT11 release through dentin of different thicknesses, correlating the depth of surface protection (TGA/Microscopy) with cellular safety limits. If limited penetration of LDT11 into the deeper dentin layers is confirmed, that may help protect the pulp tissue from exposure to high concentrations of the compound. Additional investigations using complex co-culture models with human dentin substrates are needed to determine whether the compound acts through calcium ion chelation or other chemical mechanisms. Further studies should also focus on evaluating the effects of LDT11 on the physical and mechanical properties of dentin after treated with LDT11. Moreover, in vivo studies assessing efficacy and safety are necessary to support its translational potential. Finally, this study may serve as a reference for the characterization of new materials with the potential to biomodify and preserve the dentin collagen matrix.

## Supplementary Information

Below is the link to the electronic supplementary material.

Supplementary Material 1

## Data Availability

The data that support the findings of this study are available from the corresponding author upon reasonable request.
