# Supplementary material for: Saturated anacardic acid preserves root dentin collagen under cariogenic challenge
Source: Clin Oral Investig. 2026 Sep 28;30(10):484. doi: 10.1007/s00784-026-07178-y (PMC13619660; doi:10.1007/s00784-026-07178-y)
Supplement: Supplementary file 1 [file 784_2026_7178_MOESM1_ESM.docx]

**Supplementary material**

**Materials and Methods**

**Sample Preparation**

**Supplementary Figure 1 -** Specimen preparation for FTIR-ATR experiment: bovine incisors were sectioned to obtain two dentin specimens per root (≈500 µm thickness, 8 mm width) from mesial and distal regions, excluding the root canal and cementum. Created using <https://BioRender.com>

**Supplementary Figure 2 –** Sample preparation for Picrosirius and TGA experiments. Root dentin discs were obtained by sectioning with a double-sided diamond disc. The apical region and the 2 mm below the cement-enamel junction were discarded. The samples had dimensions of 4 mm in height (Experiment 1 – Picrosirius) and 1 mm in height (Experiment 2 – TGA) × 5–6 mm in diameter. Created using <https://BioRender.com>

**Supplementary Figure 3 -** Details regarding the chemical design and structure of (a) LDT11 and (b) LDT409.

**Supplementary Figure 4 -** Pretreatment of dentin with LDT11, LDT409, or distilled water. Specimens were demineralized with 10% phosphoric acid for 30 min, rinsed, and immersed in the respective treatment solutions for 72 h at 4 °C, followed by rinsing to remove unbound compounds. Created using <https://BioRender.com>

**Supplementary Figure 5 -** *In vitro* cariogenic model for the formation of carious lesions in bovine root dentin specimens. (A) Culture and inoculation of *Streptococcus mutans* UA159 on dentin samples. (B) Sucrose cycling to simulate a cariogenic environment. Specimens were incubated at 37 °C under microaerophilic conditions for 7 days. Subsequently, samples were transferred to fresh BHI medium, and sucrose cycling was initiated: the medium was supplemented with 1% sucrose during the exposure phase (6 h) and replaced with pure BHI during the non-exposure phase (24–48 h). The cariogenic challenge lasted 7 days, including 3 days of sucrose cycling. Created using <https://BioRender.com>

**Supplementary Figure 6 -** Example of image processing performed using ImageJ software. (A) Picrosirius-stained image corresponding to Group 1, Sample A; (B) RGB color deconvolution (green, blue, and red channels); and (C) binarization of the blue channel into black and white, allowing demarcation of the root dentin and quantification of the residual collagen area.
